# A dietary strategy for the management of artemether-lumefantrine-induced cardiovascular and renal toxicity

**DOI:** 10.1186/s12906-016-1334-3

**Published:** 2016-09-06

**Authors:** Isaac Julius Asiedu-Gyekye, Mahmood Abdulai Seidu, Banga Benoit N’guessan, Samuel Frimpong–Manso, Joseph Edusei Sarkodie, Samuel Adjei, Schevadnazy Kutu, Joseph Osei-Little, Alexander Kwadwo Nyarko, Philip Debrah

**Affiliations:** 1 Department of Pharmacology and Toxicology, College of Health Sciences, University of Ghana School of Pharmacy, P. O. Box LG 43, Legon, Ghana; 2 Department of Medical Laboratory Sciences, College of Health Sciences, School of Biomedical and Allied Health Sciences, Legon, Ghana; 3 Department of Pharmaceutical Chemistry, College of Health Sciences, University of Ghana School of Pharmacy, Legon, Ghana; 4 Department of Pharmacognosy and Herbal Medicine, College of Health Sciences, University of Ghana School of Pharmacy, Legon, Accra Ghana; 5 Department of animal experimentation unit, College of Health Sciences, Noguchi Memorial Institute for Medical Research, Legon, Accra Ghana; 6 Department of Pharmaceutics and Microbiology, College of Health Sciences, University of Ghana School of Pharmacy, Legon, Ghana

**Keywords:** Cocoa, EDXRF, Artemether-Lumefantrine, Lipid profile, Renal function test, Histopathology

## Abstract

**Background:**

Unsweetened natural cocoa has antimalarial properties. Unsweetened natural cocoa powder (UNCP), obtained as a result of the removal of cocoa butter from a cocoa bean protects against malaria episodes. Cocoa powder, which is prepared after removal of the cocoa butter, contains about 1.9 % theobromine and 0.21 % caffeine. Concomitant consumption of cocoa and artemether/lumefantrine (A/L) is a common practice in Ghana, West Africa. This study seeks to determine the elemental composition of UNCP and its protective effect on the heart and kidney against (A/L) administration.

**Methods:**

Energy dispersive x-ray fluorescence spectroscopy was used to detect the quality and quantity of the elemental composition in UNCP. Thereafter, 30 nonmalarious male guinea pigs were divided into five groups of six animals each. One group was administered with 75 mg/kg body weight A/L only and another group distilled water (control group). The rest received 300 mg/kg, 900 mg/kg and 1500 mg/kg body weight UNCP for 14 days orally and A/L for the last 3 days (ie day 11 to day 14). Biochemical and histopathological examinations were carried out after euthanisation of the animals.

**Results:**

A total of thirty-eight (38) micro and macro elements were detected with the ED-XRF. Macro elements like sodium (Na), magnesium (Mg), aluminium (Al), phosphorus (P), chlorine (Cl), potassium (K), calcium (Ca), manganese (Mn) and iron (Fe) and micro elements like chromium (Cr), copper (Cu), zinc (Zn), arsenic (As), and lead (Pb) were identified and evaluated. Biochemical analysis revealed increases in HDL levels (*p*>0.05) while there were decreases in LDL levels (*p*>0.05), creatine kinase and AST levels (*P*<0.05) in animals that received UNCP compared to A/L only administered group. Urea levels reduced significantly by 53 % (*p*<0.05) in group that received 1500 mg/kg UNCP. Histopathological examinations of the heart and kidney buttressed the protective effects of cocoa administration.

**Conclusion:**

The percentage of recommended daily allowance of UNCP for chromium is 3750 % for men and 5250 % for women while % RDA for copper corresponds to 103.6 % in both sexes. UNCP proved to possess cardioprotective and renoprotective potential during artemether-lumefantrine administration.

## Background

Artemether-lumefantrine (A/L) is one of the approved fixed-dose artemisinin-based combination therapies (ACTs) that serves as the drug of choice for treatment of uncomplicated malaria in Ghana. It is commonly dispensed as an over-the-counter drug. Current trend of research is geared towards increasing the dose currently in use to prevent drug resistance [1]. A/L administration, however, generates free radicals that has the potential of causing cellular damage with evidence of both cardiotoxic, renal toxicity and other organ toxicity [1, 2]. The toxic potentials of A/L have been well reported in both humans [1, 2] and animal experiments using guinea-pigs [3–5].

While cardiovascular and renal diseases deaths account for more than 75 % in low and middle-income countries, attempts are being made to use natural products and life styles to help curb this menace [6, 7].

Cocoa, a regular diet in Ghana, and which contributes about 28 % to Ghana’s foreign exchange is known to play a major role in cardiovascular and renal health [7, 8]. Its use is often limited by the presence of heavy metals [9, 10]. The chemical components of cocoa have been well investigated using various methods [11–13]. Cocoa powder, which is prepared after removal of the cocoa butter, contains about 1.9 % theobromine and 0.21 % caffeine [14]. Most natural cocoa powder in Ghana is sweetened. The polyphenols of unsweetened natural cocoa powder (UNCP) have proven to be very vital sources of antioxidants [2]. Regular intake of unsweetened natural cocoa powder as a beverage has immense health benefits including both cardiovascular and neurodegenerative disorders, reduces platelet aggregation and improves lipid profile [9, 15, 17]. There have been reports on cocoa being used as diet mediated malaria prophylaxis, where regular intake of cocoa powder as a beverage has been associated with reduction in the incidence of episodic malaria [18]. Research has also confirmed the potential antiplasmodial activity of different fractions especially the non-polar solvent fractions (chloroform, ethylacetate and petroleum ether) of cocoa. Thus UNCP has measurable direct in vitro inhibitory effect on *P. falciparum* and support the anecdotal reports of its ability to prevent malaria as a result of its regular intake as a beverage [19, 20]. Irrespective of its many advantages, very high levels of cocoa intake could be deleterious to health, an effect believed to be caused by (−) epigallocatechin-3-gallate, a component of polyphenols in cocoa that act as pro-oxidant and is also cytotoxic to cells [21–23]. Simultaneous consumption of cocoa beverage during antimalarial treatment with A/L is expected to have dual benefits such as rapid clearance of the malaria parasites as well as amelioration of A/L-induced toxic injury to heart and kidneys.

The use of natural antioxidants such as found in cocoa could be beneficial in rectifying such damage in humans [16]. Therefore, cocoa and its products come in handy in the search for natural remedies that may offer cardiovascular and renal protective effects against high dose A/L induced organ toxicity.

This study determines the major elemental composition of significant relevance in cardiovascular and renal disorders, biochemical and histopathological changes that occur during A/L administration following prophylactic treatment with UNCP in experimental animals. The study also aimed at assessing whether UNCP will worsen or is able to prevent some common cardiovascular and renal side effects associated with the use of A/L.

## Methods

### Preparation of UNCP solution

Calculated amount (9.6 g) of Brown Gold Natural Cocoa Powder from Hords Company Ltd, (Batch number BT620IT) registered with the Ghana Food and Drugs Authority (FDA/DK06-070) was dissolved in warm distilled water (40 ml) with stirring making a concentration of 240 mg/ml (of the UNCP). The preparation was then administered to the animals via oral gavage based on their individual body weights. Fresh sample of cocoa powder extract was prepared daily for administration.

### Phytochemical analysis

Phytochemical analysis was conducted to determine the various constituents in the UNCP according to Harborne [24]. A fresh sample of UNCP was prepared each day of administration by dissolving 1 g in 1 mL de-ionized water.

#### Saponin test

About 0.5 g of UNCP was added to water in a test tube. The test tube was shaken to observe foam formation.

#### Tannins test

About 0.5 g of UNCP was dissolved in 80 % of aqueous methanol (10 cm^3^). Freshly prepared iron (III) chloride solution was added and colour change was observed.

#### Alkaloid test

About 0.1 g of the UNCP was added to 2 M HCl, stirred, warmed and filtered. The filtrate was divided into three test tubes. Draggendorff’s reagent, Mayer’s reagent and Wagner’s reagent were added, respectively, to each test tube. The colourations were observed.

#### Flavanoids test

About 0.1 g of UNCP was added to 80 % ethanol (15 cm^3^). To the filtrate was added magnesium turnings followed by concentrated HCl (0.5 cm^3^), and observed for colour changes within 10 min.

#### Cardiac glycoside test

About 0.5 g of UNCP was dissolved in chloroform (2 cm^3^) in a test tube after which concentrated sulphuric acid was carefully added down the side of the test tube to form a lower layer.

### Energy dispersive macro and micro element measurements

The UNCP was sieved using sieve of 180 μm. Energy dispersive X-ray (ED XRF) was used for simultaneous analysis and measurement of the elemental content of the UNCP. Spectro x-lab 2000 spectrometer enhanced with three-axial geometry to reduced background noise due to radiation polarization. The monochromatic radiations emitted from the X-ray tube were applied to excite the atoms of the sample. This spectrometer is equipped with Rh anode small detector and 400w Pd x-ray tube, Be end window, a Si (Li) detector, an HOPG (high oriented pyrolitic graphite) as a BARKLA polarizer (Al, Mo and Co) as secondary target. Combination of these different targets gave a typical detection limit for eight elements (Si, Al, Mg and Na) in the range of 25–50 ppm and 1–5 ppm for heavy metals. The spectrometer is factory calibrated using a number of internationally recognized standards [25, 26]. Besides, the Recommended Daily Allowance (RDA) of each element as provided by literature and WHO was also noted and percentage % RDA noted [27, 28].

### Experimental animals and Husbandry

Thirty (30) non-malarious male guinea pigs weighing 300 g–450 g were obtained from the Noguchi Memorial Institute for Medical Research, University of Ghana, Legon and divided into 5 groups of 6 animals randomly selected. The animals were allowed to acclimatize for 1 week in a well-ventilated room, maintained at a room temperature of 22.00 ± 1.00 °C and relative humidity of 60 ± 1 % and exposed to a natural daily photoperiodicity of 12 h light-dark cycle. The guinea pigs were provided with autoclaved sankofa goat and sheep pellet diet from Ghana Agro Food Company (GHAFCO), standard rodent feeds and water ad libitum. Spontaneous behaviors of all guinea pigs were observed in cages before experimental procedures were carried out. Animals used in this study were handled in accordance with the international guidelines for Care and Use of Laboratory Animals [29]. No animals showed signs of illness before the experiments. The study protocol was approved by the departmental ethical and protocol review committee and the Noguchi Memorial Institute for Medical Research Institutional Animal Care and Use Committee with protocol approval number 2013-01-3E.

### Preparation of A/L solution

A concentration of 20 mg/ml of artemether/lumefantrine (or coartem®) from Novartis Company Ltd (with reference to artemether), was prepared and administered to the guinea pigs in the UNCP treated groups at a dose of 75 mg/kg body weight daily for 3 days via oral gavage. This was done according to Osonuga, et al. [30] and Aprioku, 2012 [31]. Dosage was calculated with reference to the dose of artemether in the drug combination. To achieve this, seventy (70) tablets of Novartis coartem® dispersible tablets (20/120 mg) which is equivalent to 1400 mg of artemether, was dissolved in 70 ml of distilled water and stirred until completely homogenous.

### Administration of drugs

Guinea pigs were divided into 5 groups of 5 animals each.

Group I-Vehicle control ‘CTRL’ (distilled water for 14 days).

Group II-Negative control ‘COART’ (75 mg/kg A/L only last 3 days).

Group III-300 mg/kg UNCP (14 days) + A/L 75 mg/kg (12^th^–14^th^ day).

Group IV-900 mg/kg UNCP (14 days) + A/L 75 mg/kg (12^th^–14^th^ day).

Group V-1500 mg/kg UNCP (14 days) + A/L 75 mg/kg (12^th^–14^th^ day).

During the first 14 days of dosing, animals in groups III, IV, and V were administered prophylactic doses of the cocoa powder at 300 mg/kg, 900 mg/kg and 1500 mg/kg body weight respectively, as used in other studies [14] for 14 days against A/L administration. Animals in group I were given distilled water ie vehicle control group (VCG) for 14 days with group II receiving 75 mg/ kg A/L ie negative control group (NCG) for the last 3 days within the 14 day period. Drugs were administered by oral gavage. The weights of the animals were taken weekly and the doses administered adjusted accordingly.

In all cases, fresh solutions of UNCP and A/L were prepared before each dosing. The present study was performed according to international rules considering animal experiments.

### Biochemical assays

Animals were sacrificed after 14 days of drug treatments. Blood samples were collected into plain gel tubes, allowed to clot, centrifuged for 15 min at 3,000 rpm and sera removed and stored at-20 °C until used. Sera obtained were assayed for biochemical parameters, namely, total cholesterol, triglycerides, high density lipoproteins (HDL), low density lipoproteins (LDL), very low density lipoproteins (VLDL), creatine kinase (CK) and aspartate transferase, creatinine, urea and blood electrolytes [30, 31]. These were measured using the Selectra Junior Autoanalyser (Vital Scientific BV, Version 04, Netherlands).

### Histopathology

Euthanized guinea pigs were dissected and their hearts and kidneys were removed. The tissues were preserved in 10 % buffered formalin. The tissues were embedded in paraffin wax, sectioned at 4 μm thickness and stained with hematoxylin-eosin. Histological slides of the study animals were evaluated alongside those from the two controls groups using a light microscope. For each group of guinea pigs, a total of 30 photomicrographs were taken at magnification of × 40.

### Data analysis

Results were expressed as mean ± SEM. Data was analysed using one-way analysis of variance, followed by Newman-Keuls multiple comparison test. Values of *p*˂0.05 were considered statistically significant. Dunnet Multiple Comparison Test was used in the analysis of the nitric oxide levels.

## Results

### Phytochemical analysis

Phytochemical analysis of unsweetened natural cocoa powder showed the presence of saponins, alkaloids, flavonoids and cardiac glycosides.

### Macro and micro element analysis

In using UNCP as a beverage, general recommendation is 2–3 teaspoonful of the powder to be stirred in hot water. A teaspoon corresponds to almost five grammes (5 g). Thus Two (2) or three (3) teaspoonful comes to 10 or 15 g respectively, averaging 12.5 g of UNCP.

Comparing these values to the Recommended Daily Allowance (RDA) of each element, the estimated percentage of RDA as supplied by UNCP to consumers is determined.

The level of elements in UNCP extrapolated to literature RDA values (ie % RDA of UNCP) in both men and women are as shown in the table below.

### Biochemical assays

#### Lipid profile

**Fig. 1:**
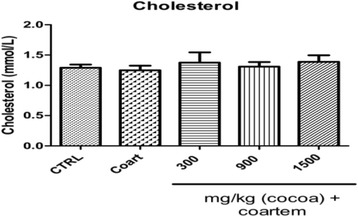
Serum cholesterol (mmol/L) concentration of male guinea pigs administered different prophylactic doses of UNCP. Values are expressed as mean ± SEM (*n* = 6). *P* values < 0.05 were considered significant

Generally, there was little change in the mean levels of cholesterol in animals in the VCG, NCG (1.242 ± 0.170 mmol/L) and those that received UNCP 300 mg/kg cocoa + A/L (1.374 ± 0.381 mmol/L), 900 mg/kg cocoa + A/L (1.380 ± 0.172 mmol/L) and 1500 mg/kg cocoa + A/L (1.388 ± 0.242 mmol/L) compared to the VCG (1.290 ± 0.119 mmol/L) (*P* < 0.05) (Fig. 1).

**Fig. 2:**
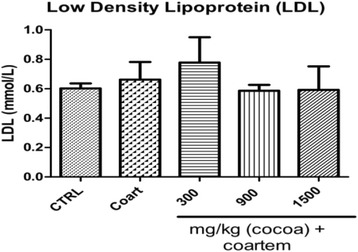
Serum LDL (mmol/) levels in male guinea pigs administered different prophylactic doses of UNCP. Values are expressed as mean ± SEM (*n* = 6). *P* values < 0.05 were considered significant

The mean serum levels of low density lipoprotein (LDL) decreased in the medium and high UNCP dose groups by 11.6 and 10.6 % (*p* < 0.05), respectively compared to the NCG (0.662 ± 0.269 mmol/L) (Fig. 2).

**Fig. 3:**
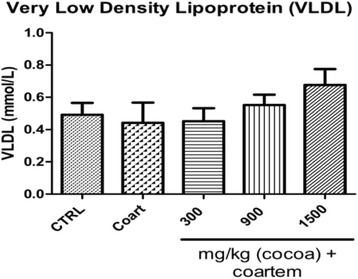
Serum VLDL (mmol/l) levels in male guinea pigs administered different prophylactic doses of UNCP. Values are expressed as mean ± SEM (*n* = 6). *P* values < 0.05 were considered significant

**Fig. 4:**
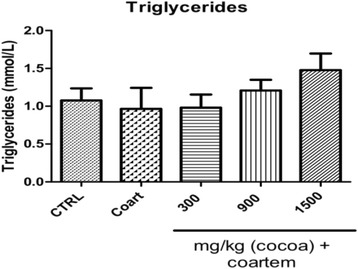
Serum triglyceride (mmol/L) levels in male guinea pigs administered different prophylactic doses of UNCP. Values are expressed as mean ± SEM (*n* = 6). *P* values < 0.05 were considered significant

Serum levels of both VLDL (Fig. 3) and triglycerides increased as the dose of administered UNCP increased. Triglyceride changes were as follows: controls (1.075 ± 0.360 mmol/L), A/L administered group (0.966 ± 0.619 mmol/L), 300 mg/kg UNCP (0.980 ± 0.391 mmol/L), 900 mg/kg UNCP (1.208 ± 0.317 mmol/L), 1500 mg/kg UNCP (1.478 ± 0.487 mmol/L) (*P* < 0.05) (Fig. 4).

**Fig. 5:**
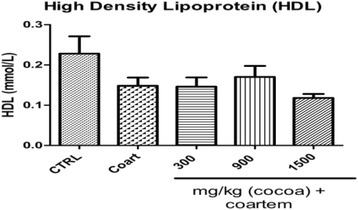
Serum HDL (mmol/L) levels in male guinea pigs administered different prophylactic doses of UNCP. Values are expressed as mean ± SEM (*n* = 6). *P* values < 0.05 were considered significant

With regard to the mean serum levels of high density lipoprotein (HDL), there was a 12.9 % (*P* < 0.05) increase in the group that received 900 mg/kg UNCP compared to the NCG 0.148 ± 0.046, (*P* < 0.05), (Fig. 5).

**Fig. 6:**
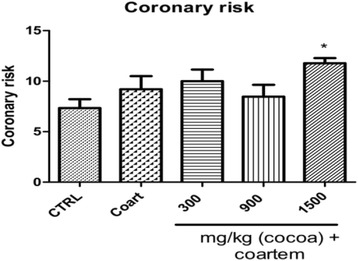
Coronary risk ratio in male guinea pigs administered different prophylactic doses of UNCP. Values are expressed as mean ± SEM (*n* = 6). *P* values < 0.05 were considered significant

The levels of coronary risk was high (11.778 ± 1.167) in animals that received 1500 mg/kg UNCP and low (8.470 ± 2.624) in the animals that received 900 mg/kg UNCP compared to the NCG (9.08 ± 2.894, *P* < 0.05) (Fig. 6).

##### AST levels

**Fig. 7:**
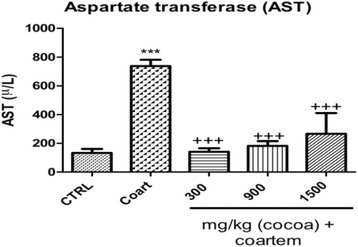
Serum aspartate transferase (μ/L) levels in male guinea pigs administered different prophylactic doses of UNCP. Values are expressed as mean ± SEM (*n* = 6). *P* values < 0.05 were considered significant

The mean serum levels of AST in the 300 mg/kg, 900 mg/kg and 1500 mg/kg UNCP administered groups were 140.8 ± 55.65U/L (80.9 %), 182 ± 73.8U/L (75.3 %), 266.6 ± 321.0U/L (63.9 %) respectively compared to the A/L administered group (737.6 ± 100U/L) (Fig. 7).

##### Creatine kinase

**Fig. 8:**
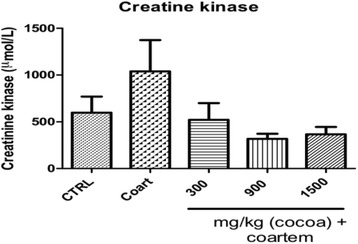
Serum creatine kinase (μmol/L) levels in male guinea pigs administered different prophylactic doses of UNCP. Values are expressed as mean ± SEM (*n* = 6). *P* values < 0.05 were considered significant

The mean levels of CK in VCG (598.0 ± 382.425 μmol/L), NCG (1039.0 ± 749.494 μmol/L) were significantly different. The groups that received 300 mg/kg UNCP), 900 mg/kg UNCP and 1500 mg/kg UNCP had their CK as follows: 552.2 ± 399.968 μmol/L, 318.5 ± 122.516 μmol/L and 366.8 ± 174.921 μmol/L respectively (Fig. 8). The LD, MD and HD cocoa groups hence reduced the creatine levels by 46.9, 69.3 and 64.7 % respectively (*P* < 0.05).

### Renal function Test

**Fig. 9:**
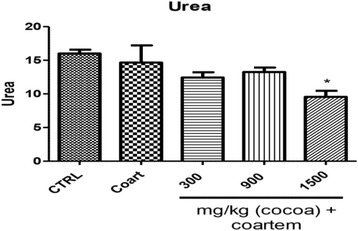
Changes in urea (μmol/L) levels during a 14- day administration of UNCP in male guinea pigs followed by a 3-day coartem® administration. Values are expressed as mean ± SEM, *n* = 6. The differences among the mean were analyzed using one-way ANOVA followed by Neuman-keul’s post hoc analysis. Vertical bars represent Mean ± SEM of Urea levels on various animal groupings. Values are considered significant when *P < 0.05

Urea was reduced by 53 % in 1500 mg/kg when compared to the VCG (*P* < 0.05). Groups 3 and 4 reduced urea by 14 and 10.64 % when compared to the VCG (Fig. 9).

**Fig. 10:**
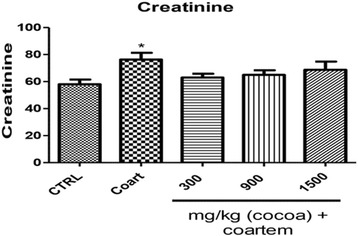
Changes in Creatinine levels (μmol/L) during a 14- day administration of UNCP in male guinea pigs followed by a 3-day coartem® administration. Values are expressed as mean ± SEM, *n* =6. The differences among the mean were analyzed using one-way ANOVA followed by Neuman-keul’s post hoc analysis. Vertical bars represent Mean ± SEM of creatinine count on various animal groupings. Values are considered significant when *P < 0.05)

Creatinine level significantly increased by 24.08 % in NCG compared to VCG. creatinine level decreased by 21.27, 17.54 and 11.05 % in Groups 3, 4 and 5 respectively when compared to group 1 (*P* < 0.05) (Fig. 10).

Sodium, potassium and chloride levels remained relatively unchanged in all groups as compared to the controls that received distilled water only.

### Histopathological Examination

The figures below show photomicrographs of myocardial tissues of animals from the different experimental groups. Sections of cardiac muscle of animals that received A/L 75 mg/kg only showed patchy areas of congestion, oedema, extensive nuclear and tissue degeneration leading to loss of microstructure of myocardial tissues. In contrast, sections of cardiac muscle of the control group and animals that received 300 mg/kg UNCP retained the normal branching of myocardial cells characteristic of normal myocardial tissue histology. Similarly, myocardial tissue sections of animals that received 1500 mg/kg largely showed normal cardiac tissue histomorphology. There was very little in terms of deterioration or inflammation to report. Significantly, there was evidence of ongoing tissue necrosis in a section of myocardial tissue of one of the animals that received 900 mg/kg UNCP. There was loss of cells and nuclei in the region pointed out. In addition, the region appeared intensely stained indicating the presence of dead cells.

## Discussion

Phytochemicals are extensively studied for the treatment of different ailments [32–34]. The pharmacological assessment of UNCP was incomplete without evaluation of its phytochemical profile. Therefore, in this study UNCP was examined for the presence of different phytochemicals (flavonoids, tannins, alkaloids, saponins, terpenoids and glycosides). The above study has shown that, UNCP contains flavonoids, alkaloids, tannins, saponins, terpenoids and glycosides which is consistent with previous findings [34]. These components have been found to play significant role in enhancing cardiovascular and renal functions.

Serum levels of total cholesterol, LDL cholesterol and TG did not change significantly during both A/L and UNCP administration. It must however be noted that lipid profile including cholesterol in general takes considerable time to show significant changes even with cholesterol lowering agents. Thus, the 14-day administration of cocoa may not have been long enough to produce significant changes in serum cholesterol and TG levels in the experimental animals. The lipid profile concurs partly with observations from a previous study that showed that short-term supplementation with cocoa products was associated with a decrease in LDL cholesterol, but had no significant effect on total cholesterol and HDL cholesterol compared with controls, an effect likely to be dependent on the amount of cocoa being consumed [35].

Proteins, cholesterol and TG in varying amounts are important components of lipoproteins of which VLDL has the highest amount of TG. It was also observed that serum VLDL levels appeared not to have been significantly affected by the administration of UNCP. This might explain the similarity in the nature of the graphs for serum TG and VLDL (Figs. 7, 8 and 9).

CK or creatine phosphokinase is a marker of damaged tissues that are rich in CK. Increases in CK levels are also most often as a result of myocardial injury [36, 37]. The study showed that animals that received 900 mg/kg bwt + A/L had 69.3 % reduction in serum CK showing the greatest mitigating activity against coartem toxicity (Fig. 1). Since CK is also concerned with the conversion of creatine to produce phosphocreatine and adenosine diphosphate, it might also protect or enhance myocardial bioenergetics [36, 37].

Further, we assessed the coronary risk ratio which is an important indicator of cardiovascular health. Coronary risk ratio in the high dose (1500 mg/kg cocoa + A/L) group was high compared to the controls (Fig. 10). This observation agrees with findings that although cocoa possesses many benefits, intake at very high levels could be deleterious to health, an effect believed to be caused by (−) epigallocatechin-3-gallate [21–23].

**Fig. 11:**
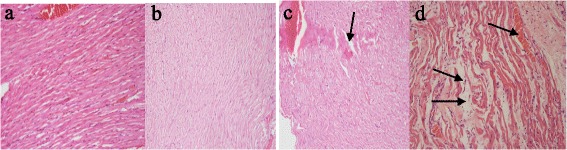
A representative section of the cardiac muscle (**a**) that retained the normal branching of myocardial cells characteristic of normal myocardial tissue histology (VCG and 900 mg/kg UNCP) (**b**) that showed very little in terms of deterioration or inflammation (1500 mg/kg UNCP) (**c**) with evidence of section of the heart with ongoing tissue necrosis in a section of myocardial tissue of one of the animals that received 300 mg/kg UNCP. The region appeared intensely stained indicating the presence of dead cells (arrowed) (**d**) showing patchy areas of congestion, oedema, extensive nuclear and tissue degeneration leading to loss of microstructure of myocardial tissues (arrowed). This was observed with animals that received 3 day A/L (75 mg/kg) only (NCG)

Aspartate transferase is an enzyme distributed mostly in the heart followed by the liver and skeletal muscles. High serum aspartate transferase values are hence indicative of cellular injury and may present in myocardial disease, shock, hypoxia, among others. Administration of distilled water + A/L significantly increased the serum levels of aspartate transferase which were significantly reduced in all animals administered unsweetened natural cocoa powder extract. These observations are corroborated by histopathological examination of the myocardial tissues of the guinea pigs (Fig. 11a). The results indicate that tissue sections from animals receiving only A/L 75 mg/kg showed evidence of inflammation and degeneration of the myocardial tissue (Fig. 11b), which buttresses the biochemical results obtained. Sections of myocardial tissue of animals administered UNCP extract largely exhibited normal cardiac tissue structure except those of animals that received 900 mg/kg UNCP where there was a single case observed with suspected ongoing tissue necrosis at the initial stages. Similar observations of the cardioprotective effect have also been made by other researchers [36–38].

According to Table 1, the elemental composition of UNCP showed the presence of sodium, potassium, calcium and magnesium believed to play major roles in the pathophysiology of cardiovascular disorders [30, 38, 39]. Furthermore, element such as magnesium, zinc, copper, and chromium are known to be involved in cellular bioenergetics [36, 37]. Thus the cardioprotective effects of UNCP may be attributed to the high content of these elements. Extrapolating animal dosage to humans and taking into account, the body surface area (BSA), then the human equivalent dose (HED) of 1500 mg/kg UNCP corresponds to 324.3 mg/kg HED, which is equivalent to 22701 mg per 70 kg average human weight (ie 22.70 g of UNCP) daily (equivalent to 9 teaspoonful daily) [40, 41]. For UNCP, the percentage RDA values for chromium for men and women is 3750 and 5250 % respectively while %RDA for copper corresponds to 103.6 % in both sexes [27, 28].

**Table 1:** Comparison of literature and calculated percentage RDA of some selected elements [29]

Element	MEAN LEVELS (mg/4g UNCP)	WHO RDA (men)	WHO RDA (women)	% RDA of UNCP (men)	% RDA of UNCP (women)
Na	2.4666 ± 0.00	1500 mg	1500 mg	0.51	0.51
Mg	33.0133 ± 0.02	420 mg	320 mg	24.60	32.30
P	64.3866 ± 0.00	700 mg	700 mg	28.70	28.70
Cl	2.3616 ± 0.00	2300 mg	2300 mg	0.32	0.32
K	149.0667 ± 0.03	4700 mg	4700 mg	10.00	10.00
Ca	11.0146 ± 0.00	1000 mg	1000 mg	3.40	3.40
Mn	0.4093 ± 0.00	2.3 mg	1.8 mg	56.50	72.20
Fe	1.0309 ± 0.00	8.0 mg	18 mg	40.25	17.90
Cr	0.4200 ± 17.44	35 μg	25 μg	3750.00	5250.00
Cu	0.2984 ± 1.71	900 μg	900 μg	103.60	103.60
Zn	0.4086 ± 0.74	11 mg	8 mg	11.60	16.00

The high content of Cu^2+^ should be of concern especially at high doses since copper has been shown to play a role in the pathogenesis of Wilson’s syndrome and liver damage. Besides other studies have shown adverse effects of high copper intake like dyslipidemia and renal dysfunction especially among diabetics [42, 43] where it also induced oxidative stress and diminished antioxidant enzymes. This effect on renal function however needs further investigation.

**Fig. 12:**
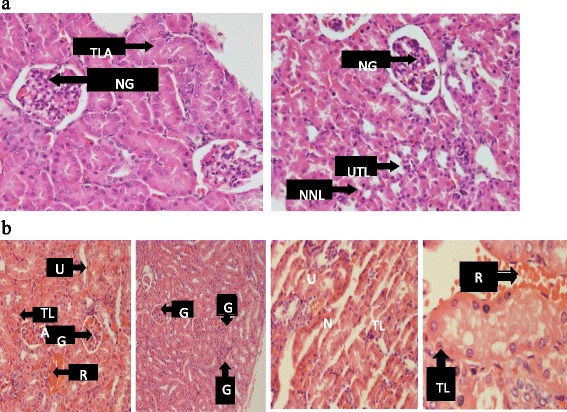
A representative section of the kidney showing (**a**) Normal nuclear lining and uncongested tubular lumen with normal glomeruli observed in the VCG and animals that received UNCP (300, 900 and 1500 mg.kg) (**b**) kidney damages observed in animals that received NCG ie A/L 75 mg/kg for 3 days (and one animal that received 1500 mg/kg UNCP). Note the severe red blood cells infiltration, congested glomeruli and tubular lining anucleasis (note left picture)

Urea levels significantly reduced in the 1500 mg/kg group as compared to the coartem® only group. Creatinine levels decreased in all the groups compared to the control group. These observed effects can be attributed to the antioxidant and nephroprotective effects of cocoa [41]. The animals that received only the 75 mg/kg coartem® group showed high levels of renal damage evidenced by the histopathological observations (Fig. 12b) and this could be due to the absence of any protective effect from the flavonoids in cocoa since they didn’t receive any UNCP administration. Animals that received 900 mg/kg UNCP showed significant renoprotective effect in the histopathological analysis (Fig. 12a). A renoprotective effect has also been reported where the activation of adenosine monophosphate-activated protein kinase (AMPK) by cocoa enriched polyphenols followed by reduction in NOX4/TGFβ-1 signaling may have a therapeutic potential in diabetic nephropathy in experimental diabetes mellitus [44–46].

Previous studies have shown increases in nitric oxide levels during UNCP administration in guinea pigs (unpublished data). Nitric oxide (NO) has been found to have renoprotective and cardioprotective effects. Thus, NO is likely to be among the mechanisms for cocoa’s protective effects. NO oxide increases associated with UNCP could be attributed to its flavonoid content [46, 47, 48]. Cocoa and flavonoid rich chocolate as well as cocoa drinks have been found to increase nitric oxide level [21, 22].

These protective effects of UNCP may be due to increased availability of antioxidants in plasma, increased plasma levels of nitric oxide mediated by constituents of cocoa such as flavonoids cocoa butter and polyphenols and also the presence of these macro and micro elements in UNCP [44, 46–49]. The above study has shown that UNCP has cardioprotective and nephroprotective potential against A/L induced toxicity. Thus the simultaneous consumption of UNCP and A/L is not likely to be deleterious to the heart and kidney but rather advantageous. It would be interesting to conduct a similar study, using malarious guinea-pigs to look at the extent and level of parasitaemia in individual test drug administration and during A/L and UNCP combination.

## Conclusion

For UNCP, the percentage RDA values for chromium for men and women is 3750 % and 5250 % respectively while %RDA for copper corresponds to 103.6 % in both sexes. Also, UNCP has cardioprotective and renoprotective potential during high dose A/L administration and thus simultaneous ingestion of A/L and UNCP may not be detrimental to the heart and kidney. However, regular consumption of large quantities of UNCP could pose health problems due to the high elemental content of copper.

### Limitation

Other cardiac and renal markers could also have been investigated. Animals infected with specific malaria parasites could have been used as a source of comparison. Other species of animals could also be used to conduct this research.

## Abbreviations

A/L: Artemether-Lumefantrine
ALB: Albumin
ALP: Alkaline phosphatase
ALT: Alanine aminotransferase
ANOVA: Analysis of variance
AST: Aspartate aminotransferase
CK: Creatinine kinase
EDXRF: Energy dispersive X-ray
FDA: Food and Drugs Authority
GAFCO: Ghana Agriculture Food Company
GC: Glomerular Congestion/ Hydropic Glomerular Degeneration
HDL: High density lipoprotein
LD: Low dose
LD_50_: Lethal dose
LDL: Low density lipoprotein
MRCI: Minimal red blood cell infiltration
NCG: Negative Control Group
NG: Normal glomeruli
NNL: Normal nuclear lining
NO: Nitric oxide
RDA: Recommended daily allowance
S-D: Sprague-Dawley
SDR: Sprague-Dawley Rats
TC: Tubular Congestion/ obliterated proximal convoluted tubular lumen
TG: Triglycerides
TLA: Tubular lining anucleasis
UNCP: Unsweetened natural cocoa powder
UTL: Uncongested tubular lumen
VCG: Vehicle Control Group
VLDL: Very low density lipoproteins

## References

[1] Efferth T, Kaina B (2010). Toxicity of the antimalarial artemisinin and its derivatives. Crit Rev Toxicol.

[2] Angus B (2014). Novel anti-malarial combinations and their toxicity. Expert Rev Clin Pharmacol.

[3] Ukekwe I. Evaluation of the subacute and delayed toxicity of artemether– lumefantrine combination in rats (Doctoral dissertation, University of Nigeria, Nsukka). 2013.

[4] Chikezie PC (2014). Comparative erythrocyte glutathione S-transferase activity profile of Non-malarious guinea pigs (*cavia tschudii*) administered pyrimethamine/sulfadoxine and artemether/lumefantrine combination therapies. Thrita.

[5] Obianime AW, Aprioku JS (2009). Comparative study of artesunate, ACTs and their combinants on the biochemical parameters of male guinea-pigs. Afr J Biotechnol.

[6] Danquah JO. Occurrence Levels Of Heavy Metals in Fermented Cocoa Beans and Cocoa Derived Products Produced in Ghana (Doctoral dissertation, University of Ghana). 2015.

[7] Arranz S, Valderas-Martinez P, Chiva-Blanch G, Casas R, Urpi-Sarda M, Lamuela-Raventos RM, Estruch R (2013). Cardioprotective effects of cocoa: clinical evidence from randomized clinical intervention trials in humans. Mol Nutr Food Res.

[8] Jumar A, Schmieder RE. Cocoa Flavanol Cardiovascular Effects Beyond Blood Pressure Reduction. J Clin Hypertens. 2015;1. 10.1111/jch.12715 PMC8031944 26514936

[9] Ford ES (2000). Serum copper concentration and coronary heart disease among US adults. Am J Epidemiol.

[10] Zhai Q, Narbad A, Chen W (2015). Dietary strategies for the treatment of cadmium and lead toxicity. Nutrients.

[11] Andres-Lacueva C, Monagas M, Khan N, Izquierdo-Pulido M, Urpi-Sarda M, Permanyer J, Lamuela-Raventos RM (2008). Flavanol and flavonol contents of cocoa powder products: influence of the manufacturing process. J Agric Food Chem.

[12] Wollgast J, Anklam E (2000). Review on polyphenols in Theobroma cacao: changes in composition during the manufacture of chocolate and methodology for identification and quantification. Food Res Int.

[13] Yang WL, Hu MH, Chen SW, Wang Q, Zhu S, Dai J, Li XZ (2015). Identification of adulterated cocoa powder using chromatographic fingerprints of polysaccharides coupled with principal component analysis. Food Anal Methods.

[14] Awortwe C, Asiedu-Gyekye IJ, Nkansah E, Adjei S (2014). Unsweetened natural cocoa Has anti-asthmatic potential. Int J Immunopathol Pharmacol.

[15] Grassi D, Desideri G, Necozione S, Lippi C, Casale R, Properzi G, Blumberg JB, Ferri C (2008). Blood pressure is reduced and insulin sensitivity increased in glucose-intolerant, hypertensive subjects after 15 days of consuming high-polyphenol dark chocolate. J Nutr.

[16] Keen C, Holt R, Oteiza P, Fraga C, Schmitz H (2005). Cocoa antioxidants and cardiovascular health. Am J Clin Nutr.

[17] Monagas M, Khan N, Andres-Lacueva C, Casas R, Urpí-Sardà M, Llorach R, Lamuela-Raventós RM, Estruch R (2009). Effect of cocoa powder on the modulation of inflammatory biomarkers in patients at high risk of cardiovascular disease. Am J Clin.

[18] Addai FK (2010). Natural cocoa as diet-mediated antimalarial prophylaxis. Med Hypotheses.

[19] Amponsah SK, Bugyei KA, Osei-Safo D, Addai FK, Asare G, Tsegah EA, Baah J, Ofori M, Gyan BA (2012). In vitro activity of extract and fractions of natural cocoa powder on *Plasmodium falciparum*. J Med Food.

[20] Amponsah SK, Dwumfour NN (2015). In vitro activity of cocoa powder extracts on some biomarkers implicated in P. Falciparum malaria pathogenesis. J Pharm Nutr Sci.

[21] Schroeter H, Heiss C, Balzer J, Kleinbongard P, Keen CL, Hollenberg NK (2006). (−)-Epicatechin mediates beneficial effects of flavanol-rich cocoa on vascular function in humans. Proc Natl Acad Sci U S A.

[22] Taubert D, Roesen R, Lehmann C, Jung N, Schömig E (2007). Effects of low habitual cocoa intake on blood pressure and bioactive nitric oxide: a randomized controlled trial. JAMA.

[23] Waltner-Law M, Wang X, Law B, Hall R, Nawano M, Granner D (2002). Epigallocatechin gallate, a constituent of green Tea, represses hepatic glucose production. J Biol Chem.

[24] Harborne JB (1998). Phytochemical methods: a guide to modern techniques of plant analysis.

[25] Anjos MJ, Lopes RT, Jesus EFO, Simabuco SM, Cesareo R (2002). Quantitative determination of metals in radish using X-ray fluorescence spectrometry. X-Ray Spectrom.

[26] Vazquez C, Barbara N, Lopez S (2003). XRF analysis of micronutrients in endive grown on soils with sewage sludge. X-Ray Spectrom.

[27] Canadian Council on Animal Care in science. Guide to the Care and Use of Experimental Animals [Internet]. 2009 [cited 2009 Sep 15]. Available from: http://www.ccac.ca/en_/standards/guidelines/additional/vol2_guinea_pigs.

[28] Nguyen H, Odelola O, Rangaswami J, Amanullah A. A review of nutritional factors in hypertension management. Int J Hypertens. 2013;1144–1150. 10.1155/2013/698940 PMC3649175 23691281

[29] Institute of Medicine (US) Standing Committee on the Scientific Evaluation of Dietary Reference Intakes (1997). Dietary reference intakes for calcium, phosphorus, magnesium, vitamin D, and fluoride.

[30] Osonuga IO, Osonuga OA, Osonuga A, Onadeko AA, Osonuga AA (2012). Effect of artemether on hematological parameters of healthy and uninfected adult Wistar rats. Asian Pac J Trop Biomed.

[31] Aprioku JS, Obianime AW (2012). Evaluation of biochemical indices following administration of artemether, halofantrine and a combination of artemether and lumefantrine in guinea pigs. J Applied Pharm Sci.

[32] Qayyum RA, Sarfraz A, Ashraf SA (2016). Phenolic composition and biological (anti diabetic and antioxidant) activities of different solvent extracts of an endemic plant (Helitropium strigosum). J Chilean Chem Soc.

[33] Ashraf A, Sarfraz RA, Rashid MA, Shahid M (2015). Antioxidant, antimicrobial, antitumor, and cytotoxic activities of an important medicinal plant (Euphorbia royleana) from Pakistan. J Food Drug Anal.

[34] Gunalan G, Subhashini R, Mahadeva RS, Sumathi PA (2010). Comparative phytochemical analysis of cocoa and green tea. Indian J Sci Technol.

[35] Jia L, Liu X, Bai YY, Li SH, Sun K, He C, Hui R (2010). Short-term effect of cocoa product consumption on lipid profile: a meta-analysis of randomized controlled trials. Am J Clin Nutr.

[36] Katrina G, Aleksander A, Susanna P, Guillermo C, Francisco V, Anne M (2012). The cocoa flavanol (−)-epicatechin exerts its cardioprotective effects by protecting myocardial bioenergetics. FASEB J.

[37] Kirch N, Ellinger S (2014). Cocoa flavanols and cardioprotective effects. Which flavanols may contribute to vascular health. Ernahrungs Umschau.

[38] Houston M (2011). The role of magnesium in hypertension and cardiovascular disease. J Clin Hypertens.

[39] Walpole SC, Prieto-Merino D, Edwards P, Cleland J, Stevens G, Roberts I (2012). The weight of nations: an estimation of adult human biomass. BMC Public Health.

[40] Reagan-Shaw S, Nihal M, Ahmad N (2008). Dose translation from animal to human studies revisited. The FASEB J.

[41] Asiedu-Gyekye IJ, Antwi-Boasiako C, Oppong S, Arthur S, Sarkodie JE. Haematological changes and nitric oxide levels accompanying artemether-lumefantrine administration in male guinea pigs: effect of unsweetened natural cocoa powder. J Intercult Ethnopharmacol. 2016;5: doi: 10.5455/jice.20160721104042. 10.5455/jice.20160721104042 PMC5061477 27757264

[42] Galhardi CM, Diniz YS, Faine LA, Rodrigues HG, Burneiko RC, Ribas BO, Novelli EL (2004). Toxicity of copper intake: lipid profile, oxidative stress and susceptibility to renal dysfunction. Food Chem Toxicol.

[43] Minnesota Department of Health (2000). Health effects of excess copper; copper in drinking water.

[44] Arts IC, Hollman PC (2005). Polyphenols and disease risk in epidemiologic studies. Am J Clin Nutr.

[45] Papadimitriou A, Peixoto EB, Silva KC, de Faria JML, de Faria JBL (2014). Increase in AMPK brought about by cocoa is renoprotective in experimental diabetes mellitus by reducing NOX4/TGFβ-1 signaling. J Nutr Biochem.

[46] Billiar R, Kim M, Tzeng E (1997). Role of NO and nitrogen intermediates in regulation of cell functions.

[47] Chamane N, Lochner A, Strijdom H (2009). Polyphenols and disease risk in epidemiologic studies. Cardiovasc J Afr.

[48] Kelm M, Rassaf T (2008). Cocoa flavanols and the nitric oxide-pathway: targeting endothelial dysfunction by dietary intervention. Drug Discov Today Dis Mech.

[49] Schinella G, Mosca S, Cienfuegos-Jovellanos E, Pasamar MÁ, Muguerza B, Ramón D, Ríos JL (2010). Antioxidant properties of polyphenol-rich cocoa products industrially processed. Food Res Int.

